# Recurrences of Bell's palsy

**Published:** 2014

**Authors:** D Cirpaciu, CM Goanta, MD Cirpaciu

**Affiliations:** * Alexandria County Emergency Hospital, Romania; ** ”Carol Davila” University of Medicine and Pharmacy, Bucharest, Romania; *** “Acad. Vasile Candea” Army's Center for Cardiovascular Diseases, Romania

**Keywords:** Bell’s palsy, recurrent facial palsy, Melkerson-Rosenthal syndrome

## Abstract

**Introduction**. Bell’s palsy in known as the most common cause of facial paralysis, determined by the acute onset of lower motor neuron weakness of the facial nerve with no detectable cause. With a lifetime risk of 1 in 60 and an annual incidence of 11-40/100,000 population, the condition resolves completely in around 71% of the untreated cases. Clinical trials performed for Bell’s palsy have reported some recurrences, ipsilateral or contralateral to the side affected in the primary episode of facial palsy. Only few data are found in the literature. Melkersson-Rosenthal is a rare neuromucocutaneous syndrome characterized by recurrent facial paralysis, fissured tongue (lingua plicata), orofacial edema.

**Purpose**. We attempted to analyze some clinical and epidemiologic aspects of recurrent idiopathic palsy, and to develop relevant correlations between the existing data in literature and those obtained in this study.

**Methods & Materials**. This is a retrospective study carried out on a 10-years period for adults and a five-year period for children.

**Results**. A number of 185 patients aged between 4 and 70 years old were analyzed. 136 of them were adults and 49 were children. 22 of 185 patients with Bell’s palsy (12%) had a recurrent partial or complete facial paralysis with one to six episodes of palsy. From this group of 22 cases, 5 patients were diagnosed with Melkersson-Rosenthal syndrome. The patients’ age was between 4 and 70 years old, with a medium age of 27,6 years. In the group studied, fifteen patients, meaning 68%, were women and seven were men. The majority of patients in our group with more than two facial palsy episodes had at least one episode on the contralateral side.

**Conclusions**. Our study found a significant incidence of recurrences of idiopathic facial palsy.

Recurrent idiopathic facial palsy and Melkersson-Rosenthal syndrome is diagnosed more often in young females.

Recurrence is more likely to occur in the first two years from the onset, which leads to the conclusion that we should have a follow up of patients diagnosed with Bell’s palsy for at least two years from the onset, especially in children’ case.

The frequency of recurrent facial palsy in children was similar to that in adults. Recurrent idiopathic facial palsy is not known enough and needs further controlled studies.

## Introduction

Bell's palsy (idiopathic facial paralysis) is caused by the acute onset of the lower motor neuron weakness of the facial nerve with no detectable cause [**1**]. 

Bell’s palsy is known as the most common cause of facial paralysis and even though it has been studied over time, no evident cause was found. Patients present to the doctor, with a complete or partial inability to move facial muscles on one side of the face. The symptoms are usually installed suddenly, the patients or the entourage notices the facial asymmetry, the patient complains of difficulties in mastication, impossibility to whistle, impossibility to close the eyelid that leads to dryness of the eye and potential eye injury. Some authors considered Bell’s palsy a vasculopathy in vasa nervorum of the facial nerve, others tried to demonstrate its viral etiology but no conclusive evidence was found. It was associated with cold exposure. Idiopathic facial palsy remains a diagnostic by exclusion.

**Fig. 1:**
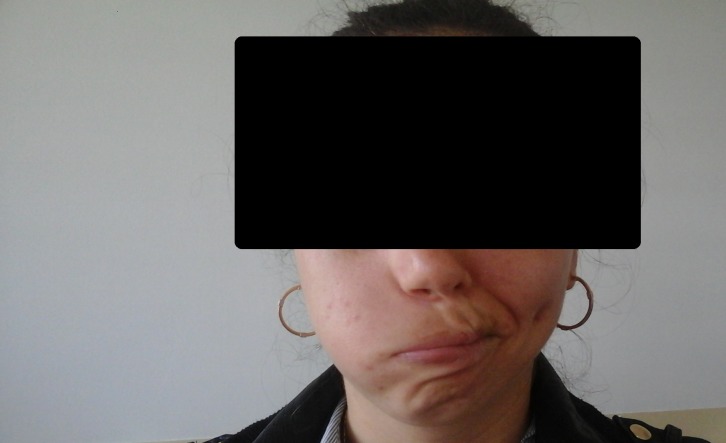
A patient with right facial palsy, dynamic asymmetry

**Fig. 2:**
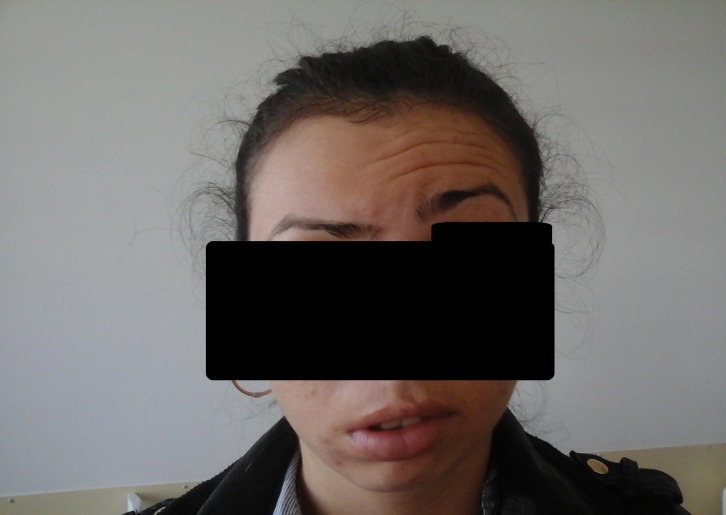
Same patient, no wrinkles on the right side of the forehead

The lifetime risk is 1 in 60, and is more common in pregnancy and diabetes mellitus [**4**]. Patients often present with facial pain or paraesthesia, altered taste and intolerance to loud noise in addition to facial droop [**4**]. It is probably caused by ischemic compression of the facial nerve within the meatal segment of the facial canal [**4**]. With a lifetime risk of 1 in 60 and an annual incidence of 11-40/100,000 population, the condition completely resolves in around 71% of untreated cases [**1**,**17**,**18**]. It is an unilateral, partial or complete paralysis of the facial nerve without the other neurological complains [**3**,**5**]. Spontaneous idiopathic facial nerve (Bell's) palsy leaves residual hemifacial weakness in 29%, which is severe and disfiguring in over half of these cases [**4**].

The clinical trials performed for Bell’s palsy, have reported some recurrences, ipsilateral or contralateral to the side affected in the primary episode of the facial palsy. Only few data are found in literature.

Melkersson-Rosenthal is a rare neuromucocutaneous syndrome [**14**] characterized by recurrent facial paralysis, fissured tongue (lingua plicata), orofacial edema. Some authors suggested a family history and a genetic predilection. Fissured, reddish-brown, swollen, nonpruritic lips characterize the orofacial swelling or firm edema of the face, the fissured tongue is seen in one third to one-half of the patients [**15**]. We may not encounter all the three symptoms. The facial palsy in this syndrome can commence even in childhood and it may precede or coincide with the onset of edematous attacks. The edema usually affects the upper lip and cheeks [**22**].

The histological evidence is not necessary for the diagnosis of Melkersson-Rosenthal syndrome, while coronary high-resolution CT reconstruction of temporal bone and food allergen detection may be beneficial [**12**,**13**].

For some time, idiopathic facial palsy was considered a condition of adults only, but this theory proved wrong because facial palsy was encountered in children as well.

There are fewer studies of children, than of adults. Some authors say that the prognosis is more favorable in children than in adults [**12**].The majority of children with Bell's palsy have a complete resolution of the facial weakness [**11**].

About the treatment:

Many clinical trials showed a significant benefit of treating Bell's palsy with corticosteroids. A recent study investigated the benefit of intratympanic steroid injection combined with systemic steroid and antiviral agents, but a complete recovery rate was not different from the group that only received the medication [**8**,**9**].

There is growing evidence that steroids are not beneficial for the treatment of pediatric patients with Bell's palsy. A retrospective longitudinal study examining notes of 100 children was published in “Journal of child neurology”, October 2014. Of the 79 patients diagnosed with Bell's palsy, all recovered with or without steroid treatment, with no statistically significant difference in symptoms duration [**10**].

High quality evidence showed no significant benefit from anti-herpes simplex antiviral compared with placebo in producing a complete recovery from Bell's palsy. Moderate quality evidence showed that antivirals were significantly less likely than corticosteroids to produce complete recovery [**6**].

There was no high quality evidence to support a significant benefit or harm from any physical therapy for idiopathic facial paralysis. There was low quality evidence that tailored facial exercises that could help improve the facial function, mainly for people with moderate paralysis and chronic cases. There was low quality evidence that facial exercise reduced sequelae in acute cases [**2**]. Hyperbaric oxygen therapy in a trial was applied in moderate to severe Bell's palsy, and the result suggested a benefit, but there was not enough evidence to support this therapy.

A number of studies published in China have suggested acupuncture is beneficial for facial palsy but the quality of the included trials was inadequate to allow any conclusion about the efficacy of acupuncture [**7**].

It seems that the only therapy that works is the therapy with steroids.

Patients with recurrent facial palsy and Melkersson-Rosenthal syndrome received almost the same treatment as those with Bell’s palsy.

In a study of seventy-two patients with recurrent facial palsy who were treated with the same treatment approach as 1,185 patients with general facial palsy, the recovery rate was lower in patients with recurrent than with primary facial palsy, but there were no significant differences in recovery rates between recurrences on the ipsilateral and contralateral sides [**16**]. The prognosis of patients with recurrent facial palsy was associated with the initiation of treatment within 7 days [**16**].

A study on eight patients with recurrent facial palsy in Melkersson-Rosenthal syndrome, treated with transmastoid subtotal facial nerve decompression, published in “Acta oto-laryngologica” this year, had an interesting outcome. Authors found that the subtotal facial nerve decompression seemed to be effective to prevent further episodes of facial palsy and promote facial nerve recovery for recurrent facial palsy in Melkersson-Rosenthal syndrome; and also that the main inflammatory sites of recurrent facial palsy in Melkersson-Rosenthal syndrome might be the mastoid segment, tympanic segment, geniculate ganglion, and labyrinthine segment [**20**].

## Materials and Methods

• This is a retrospective study carried out on a 10-years period for adults and a five-year period for children, between January 2004 and December 2013. A number of 136 adults and 49 children were included in the study. The records of patients who have addressed “Profesor Dr. Dorin Hociota” Institute of Phonoaudiology and Functional ENT Surgery, Bucharest, Romania, between January 2004 and December 2013, the ENT Department of Alexandria County Emergency Hospital, Romania, between January 2009 and December 2013, and the ENT Department of “Marie Curie” Children's Hospital, Bucharest, Romania, between 2005 and 2009, have been analyzed. We have tried to introduce as many parameters as we could in order to obtain accurate results, a part of them being shown in this paper.

Inclusion criteria:

• Patients between 4 and 70 years old with minimum one recurrence of partial or complete facial paralysis, without any specific cause were included,

• Only patients hospitalized in an ENT service were included in the study,

• The patients were divided according to demographic parameters, such as age, sex, the side affected, the number of recurrences, treatment received, related diseases, 

• Excel 2007 was used for graphics.

Exclusion criteria:

• Cases with paralysis secondary to trauma, chronic or acute otitis media, otomastoiditis, zoster oticus, cerebral stroke, or other neurological disorder or condition affecting the ear and the parotid gland, were excluded, 

• Neither patients who were not admitted, nor patients treated in a neurology department, were analyzed.

## Results

**Fig. 3:**
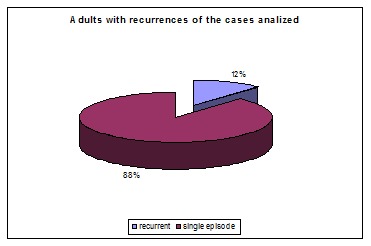
Number of adults with recurrences compared with the number adults with one single episode of facial palsy

**Fig. 4:**
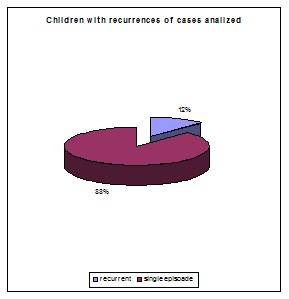
Number of children with recurrences compared with the number of children with one single episode of facial palsy

A number of 185 patients aged between 4 and 70 years old were analyzed. 136 of them were adults and 49 were children. 22 of 185 patients with Bell’s palsy (12%) had a recurrent partial or complete facial paralysis with one to six episodes of palsy. Nine of the forty-six children had recurrent facial palsy and sixteen of the one hundred and thirty-six adults with Bell’s palsy suffered recurrences. The percent of children with recurrent facial palsy was similar to the one of adults as seen in the **Table 1**, **Fig. 3** and **Fig. 4**.

**Table 1:** Number of patients from the studied group suffering from facial paralysis recurrences

	Number of idiopathic facial palsy analyzed					
	ADULTS			CHILDREN		
TOTAL	TOTAL	recurrent	single episode	TOTAL	recurrent	single episode
185	136	16	120	49	6	43

From this group of 22 cases, 5 patients were diagnosed with Melkersson-Rosenthal syndrome. All five patients were young women, four had ages between 20 and 30 years old, and one was 42 years old. Three of them had all 3 signs (recurrent facial palsy, edema of the lips or cheeks, fissured tongue), and the others had only two. One patient had a family history of fissured tongue (her mother). Another woman with Melkersson-Rosenthal syndrome, who had six attacks of facial palsy, also suffered from Basedow disease. One patient accused ipsilateral ear pain, and presented neurosensorial hearing loss to the contralateral ear, and another complained of dizziness. All five patients received treatment with steroids, vasodilators, vitamins B; in two cases, heparin was associated. One had a complete recovery when leaving the hospital.

**Table 2:** Melkersson-Rosenthal syndrome out of recurrent idiopathic facial palsy

Recurrent facial palsy	Melkersson- Rosenthal syndrome	non Melkersson recurrences
22	5	17

**Fig. 5:**
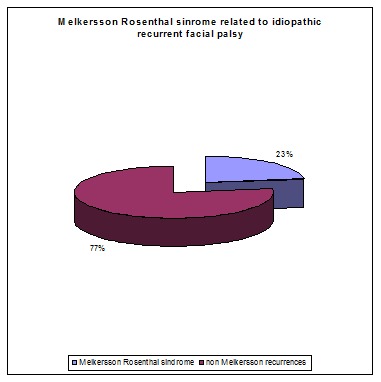
Melkersson-Rosenthal syndrome out of recurrent idiopathic facial palsy

The patients’ ages were between 4 and 70 years old, with a medium age of 27,6 years. In the age distribution, the highest incidence of patients admitted for Bell’s palsy recurrences was identified between 21 an 30 years of age, nine patients of twenty-two. The lowest incidence between the ages 51 to 60, no record being found for this category (**Table 3**). The youngest patient found in the analyzed records was of four years old and had a first episode of facial palsy at the age of one year and a half, recovered with treatment. His medical history showed that, six months after the first episode, the child suffered from meningitis. The oldest patient in the group was 63 years old with associated high blood pressure and diabetes mellitus.

**Table 3:** Distribution of recurrent facial palsy related to age

age category	Number of patients
<10 years	3
11-20 years	3
21-30 years	9
31-40 years	2
41-50 years	4
51-60 years	0
61-70 years	1
Total	22

**Fig. 6:**
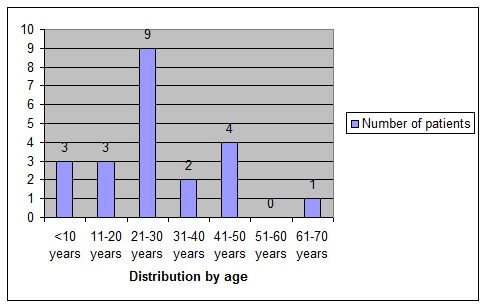
Distribution of recurrent facial palsy related to age

**Fig. 7:**
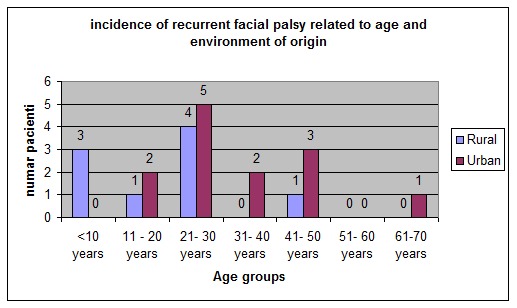
Incidence of recurrent facial palsy related to age and environment of origin

More patients from urban area were admitted as seen in **Table 4** and **Fig. 7**.

**Table 4:** Incidence of recurrent facial palsy related to age and environment of origin

age	Rural	Urban
<10 years	3	0
11-20 years	1	2
21-30 years	4	5
31-40 years	0	2
41-50 years	1	3
51-60 years	0	0
61-70 years	0	1
Total	9	13

In the group studied, fifteen patients, meaning 68%, were women and seven were men. That showed a predominance of females leading to the conclusion that females are more likely to develop another episode of facial paralysis after they were diagnosed with Bell’s palsy.

**Table 5:** Distribution according to gender of the patients with recurrences of idiopathic facial palsy

Gender distribution of recurrent facial palsy	
women	man
15	7

**Fig. 8:**
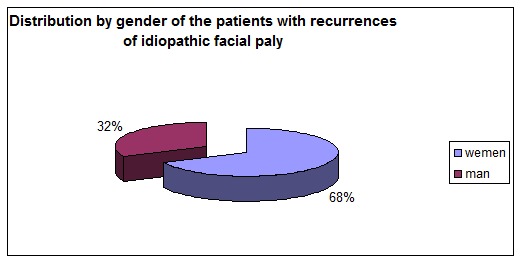
Distribution according to gender of the patients with recurrences of idiopathic facial palsy

**Fig. 9:**
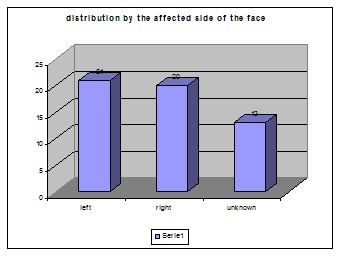
Number of facial palsy episodes right/ left side of the face

No significant differences between the affected sides of the face were found. There were 54 episodes of facial palsy in the group of the 22 patients analyzed. For 13 episodes, we did not know the affected side because records did not say or the patients could not remember (**Table 6**, **Fig. 9**).

**Table 6:** Number of facial palsy episodes related to the affected side of the face

Affected side		
left	right	unknown
21	20	13

An interesting result was that the patient with more than 3 episodes of facial palsy seemed to have a predilection side of paralysis. A patient with 4 episodes of facial palsy had three episodes on the same side and one episode on the other side of the face. A sixteen-year old boy had 4 episodes on the same side with the first episode at the age of 10. Another patient with six episodes of facial palsy had five episodes on the same side and one episode on the other side of the face. The majority of patients in our group with more than two facial palsy episodes had at least one episode on the contralateral side (The side of the first episode was considered the ipsilateral side).

**Table 7:** The side of idiopathic facial palsy recurrences

number of recurrences on the same side	number of recurrences on the contralateral side
15	7

**Fig. 10:**
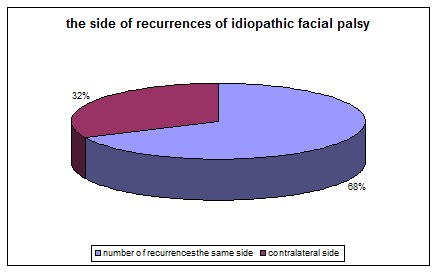
The side of idiopathic facial palsy recurrences

Regarding the number of episodes encountered in patients with recurrent facial palsy, eleven patients had two episodes, two patients had three, and two patients had four episodes of facial palsy. We found one patient with five episodes and another one with six episodes of facial palsy (**Table 8**). During the study, we have noticed that patients with more that two episodes of facial palsy had the debut in early childhood which made us think that paralysis in small children is more likely to repeat, but, in order to prove this theory, more data was needed, because adults often do not remember when the first episode occurred, and we cannot follow patients who are now children during their entire lifetime. Four patients had no record to prove the number of episodes, neither remembered that information.

**Table 8:** Number of episodes encountered in patients with recurrent facial palsy

2 episodes	3 episodes	4 episodes	5 episodes	6 episodes	unknown
11	2	2	1	1	4

**Fig. 11:**
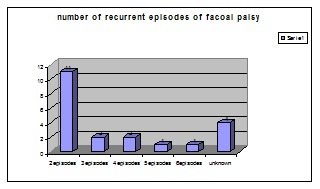
Number of episodes encountered in patients with recurrent facial palsy

Another interesting result of this analysis was that a recurrence was more likely to occur in the first two years from the debut as it is shown in **Table 9**, which led to the conclusion that a follow up of the patients diagnosed with Bell’s palsy should be done for at least two years from the onset, especially in the children case. In fourteen patients, the free interval between the first episode of facial palsy and the next one was identified. Six of them had the next episode of facial palsy during the next two years, but three patients who repeated the palsy after more than twenty years were found.

**Table 9:** Free interval between the first episode of facial palsy and the next one

years between the first episode and the second one		1- 2 years	4-5 years	6-7years	>20
Number of patients who remembered	14	6	3	2	3

During the study, we have noticed that patients who suffered many episodes of facial palsy on one side usually presented a complete facial paralysis and had a poor recovery. We could not present high quality evidence for the following observations: number of recurrences a patient suffered, the poor chance she/ he had to completely recover. This result is in concordance with the findings in literature and motivates us in searching methods to prevent recurrences. 

## Discussions

Bell’s palsy is a unilateral, acute, partial or complete paralysis of the facial nerve without other neurological complains [**3**].

Bell’s palsy in known as the most common cause of facial paralysis and even though it has been studied over time, no evident cause was found.

While patients with Bell's palsy enter the health care system with facial paresis/ paralysis as a primary complaint, not all the patients with facial paresis/ paralysis have Bell's palsy [**21**]. It is a concern that patients with alternative underlying etiologies may be misdiagnosed or have an unnecessary delay in the diagnosis [**21**].

Some authors consider Bell’s palsy a vasculopathy in vasa nervorum of the facial nerve, others tried to demonstrate its viral etiology, but no conclusive evidence was found. It was associated with cold exposure, but it also appeared in the warm seasons, and another question was raised: why is only one side affected when both left and right side are exposed. Bell’s palsy remains a diagnosis by exclusion.

Therapy has been tried in many combinations and only steroid administration seems to help.

During the study, we have noticed that patients with more that two episodes of facial palsy had the debut in early childhood which made us think that paralysis in small children is more likely to repeat, but, in order to prove this theory we needed more data, because adults often do not remember when the first episode occurred, and we cannot follow patients who are now children during their entire lifetime. The prognosis is more favorable in children than in adults [**12**]. The majority of children with Bell's palsy have a complete resolution of the facial weakness [**11**]. 

Clinical trials performed for Bell’s palsy, have reported some recurrences, ipsilateral or contralateral to the side affected in the primary episode of facial palsy. Only few data were found in literature. There are fewer studies of children than of adults. After analyzing the patient’s records, we believed that those who suffered many episodes of facial palsy on one side usually presented a complete facial paralysis and had a poor recovery. We could not present high quality evidence for the following observations: number of recurrences a patient suffered, the poor chance she/ he had to completely recover. This result is according to the findings in literature and motivates us in searching methods to prevent recurrences. The Melkersson-Rosenthal syndrome is a rare disease of unknown pathogenesis in which oligosymptomatic forms predominate [**19**]; and often the diagnostic is delayed until at least two symptoms appear. If the facial palsy is the first symptom, the patient is considered to suffer from Bell’s palsy.

We hope this study will help clinicians manage Bell’s palsy by knowing that recurrence is expected in more than 1 out of 10 patients, and will motivate them search for methods of preventing recurrences.

## Conclusions

• Our study found a significant incidence of recurrences of idiopathic facial palsy.

• Recurrent idiopathic facial palsy and Melkersson-Rosenthal syndrome are diagnosed more often in young females.

• Recurrence is more likely to occur in the first two years from the onset which leads to the conclusion that we should have a follow up of patients diagnosed with Bell’s palsy for at least two years from the onset, especially in the case of children.

• The frequency of recurrent facial palsy in children was similar to that in adults.

• Recurrent idiopathic facial palsy is not known enough and needs further controlled studies.
